# A Systematic Review of Factors Affecting Utilization of Decision Support Systems: The Interplay Between Technology, Users, and the Healthcare Environment

**DOI:** 10.1002/hsr2.72665

**Published:** 2026-06-12

**Authors:** Mohsen Khosravi, Zahra Zare, Seyyed Morteza Mojtabaeian, Reyhane Izadi

**Affiliations:** ^1^ Social Determinants of Health Research Center Birjand University of Medical Sciences Birjand Iran; ^2^ Health Human Resources Research Center, School of Health Management and Information Sciences Shiraz University of Medical Sciences Shiraz Iran; ^3^ Department of Healthcare Services Management, School of Health Management and Information Sciences Shiraz University of Medical Sciences Shiraz Iran

**Keywords:** clinical decision‐making, decision making, decision support system, delivery of health care, utilization

## Abstract

**Background and Aims:**

In the ever‐evolving domain of healthcare, Decision Support Systems (DSS) stand at the forefront of technological advancement, promising to revolutionize patient care. This systematic review investigated the factors that influence the adoption of DSS, and provides important insights into their use in healthcare services.

**Methods:**

Using the PRISMA framework, this systematic review was conducted based on articles published in the time frame of 2000–2024. A systematic literature search was conducted across PubMed, Scopus, ProQuest, and the Cochrane Library. The AACODS checklist was used for quality assessment of the included studies. Subsequently, a thematic analysis was conducted on the acquired data.

**Results:**

Eighty‐six studies were included in the research. The included studies were deemed to bear high quality and low risk of bias. The research illuminated 11 themes and 47 sub‐themes. The themes included system design and integration, user characteristics, personalization, trust, quality, utility, satisfaction and acceptability, education and awareness, involvement, settings, and ethical‐legal issues.

**Conclusion:**

The study yielded comprehensive data that addressed the research questions by presenting an extensive list of factors influencing the utilization of DSS in healthcare. The perceived importance of these factors varied, as indicated by the differing frequencies with which they were cited across the included studies. This disparity in factor importance was particularly pronounced when comparing developed and developing countries.

## 1 Introduction

In healthcare, the process of decision‐making carries significant weight, as medical practitioners are tasked with making judicious choices that carry profound implications for patients, communities, and broader national contexts. Often, these decisions must be made under conditions of limited information, resources, and expertise, yet they are expected to be executed with a high degree of precision. Given the potential for life‐altering consequences, the inherent complexities of medical decision‐making highlight its paramount importance [1].

Healthcare systems are confronted with a myriad of challenges in decision‐making. These challenges encompass the absence of standardized clinical guidelines, ineffective organizational management, regulatory conflicts, obsolete knowledge and skills, equipment scarcity, suboptimal design of systems and processes, tardy adoption of information technology innovations, inadequate consideration of patients' diverse demands and needs, personnel deficits, and hurdles in the implementation of health information systems [2, 3, 4].

The field of decision‐making within healthcare systems has witnessed substantial growth with the introduction of technologies such as artificial intelligence (AI). Multiple studies have highlighted significant advancements in the utilization of AI within decision‐making realms of healthcare systems [5, 6, 7]. AI has demonstrated considerable potential in bolstering clinical decision‐making, organizational decision‐making, and facilitating shared decision‐making within healthcare settings [6].

A Decision Support System, specifically a Clinical Decision Support System (CDSS) in the healthcare sector, is a computerized construct engineered to aid healthcare professionals in formulating clinical decisions. This is achieved by supplying pertinent clinical knowledge, patient data, and other health‐related information. The primary objective of a CDSS is to augment healthcare delivery by fortifying medical decisions with evidence‐based suggestions and patient‐specific evaluations. Studies have demonstrated that these systems are indispensable for realizing the full advantages of Electronic Health Records and are employed to facilitate the decision‐making process in the healthcare domain [4, 8, 9].

CDSS in the healthcare sector have a multitude of impacts. These impacts encompass advantages such as a decrease in the incidence of misdiagnosis, enhancement of efficiency and patient work productivity, provision of more individualized care, improvement in care quality and knowledge base, augmentation of confidence in decision‐making, betterment of prescribing behavior, and a reduction in the quantity of ordered laboratory and medical imaging tests [10].

Existing literature features several systematic reviews examining the factors that influence the utilization of DSS within healthcare organizations [11, 12, 13, 14]. However, the findings from these reviews appear to be limited in scope and, crucially, present conflicting conclusions regarding the relative importance of individual factors. This divergence is primarily evidenced by the frequency with which these factors are cited in the original research studies conducted within specific contexts. Specifically, the literature offers contradictory reports concerning the significance of both technological and organizational factors in DSS adoption within healthcare settings [11, 12, 13, 14]. This inconsistency highlights a discernible gap in the existing body of knowledge, which has not yet provided a comprehensive overview of the factors affecting DSS in healthcare organizations. This research endeavors to address this deficiency by undertaking a thorough systematic search of relevant databases. The objective is to generate a comprehensive list of factors influencing DSS adoption in healthcare, thereby offering a consolidated understanding for stakeholders and identifying the most critical factor groups for end‐users.

The objective of this study was to aggregate the results of previous research, offering a holistic perspective for stakeholders, and thereby aiding in the improvement of DSS deployment procedures within healthcare infrastructures. In this regard, the findings of this study offer significant insights into the factors influencing the utilization of DSS within healthcare centers. These insights could be leveraged by healthcare policymakers and administrators, furnishing them with the requisite data to formulate effective plans and strategies. Consequently, this would facilitate the efficient and effective implementation of such technologies within their respective organizations.

## 2 Method

### 2.1 Design

The study was organized as a systematic review conducted in 2024, following the Preferred Reporting Items for Systematic Reviews and Meta‐Analyses (PRISMA) guidelines [15].

### 2.2 Identifying the Research Question

Given the critical role of research questions in systematic review studies and the significance of a well‐crafted question during the formulation stage, it is essential to structure the research question effectively, focusing on the study's central theme. In this study, our primary interest was to identify the factors influencing the utilization of DSS in the healthcare sector. As a result, the study aimed to address the question: “what are the factors influencing utilization of DSS in healthcare?” The sub‐questions were formulated as follows:

1. What are the most prominent factors influencing the utilization of DSS in healthcare, demonstrated by the frequency of their citations within the literature?
2. What are the connections and relationships between the various groups of factors that affect the utilization of DSS in healthcare, as presented by the thematic analysis of data?

### 2.3 Search Strategy

Up to February 19, 2024, publications were systematically sought across databases, including PubMed, Scopus, ProQuest, and the Cochrane Library. The search was refined by extracting relevant keywords such as MeSH and Library of Congress Subject Headings. A preliminary search was conducted to optimize keywords, and titles, abstracts, and indexes of pertinent articles were thoroughly examined. The primary keywords for the database search were “Utilization,” “Healthcare,” and “Decision support system,” supplemented by additional keywords detailed in Table 1. The search strategy utilized Boolean operators, employing “OR” to combine words within each row and then using “AND” to combine rows, thereby enhancing search sensitivity. Manual searching on Google Scholar, based on article titles, complemented the systematic electronic search by combining the identified keywords. This process was undertaken to locate and access the full text of manuscripts available on external websites, rather than exclusively through the original journal's platform.

**Table 1:** The search strategy of the systematic review.

Concept	Description	Search term
Concept 1	Utilization	Utilize* OR engage* OR Usability OR user experience OR satisfaction OR usage OR use OR using OR adopt* OR accept* OR perspective* OR percept* OR involve* OR barrier*
Concept 2	Healthcare	Health* OR healthcare OR hospital* OR clinic*
Concept 3	Decision support system	Decision support system*

### 2.4 Inclusion Criteria and Study Selection

Following a comprehensive systematic and manual search, articles underwent an initial evaluation based on their titles and abstracts, followed by a thorough examination of their full texts. For inclusion, all English manuscripts presenting the factors influencing utilization of decision support systems in healthcare and published between 2000 and 2024 were considered. EndNote's software version 20 facilitated reference management and documented the search process. In the initial screening, two researchers (M.K., Z.Z.) independently reviewed titles. After screening the titles, the abstracts of the remaining papers were assessed by the same researchers, resulting in the removal of studies not aligning with the study's objectives. Finally, full‐text articles underwent further appraisal, and the included articles were chosen for thematic analysis.

Manuscripts were included or excluded based on the following criteria:

Inclusion:

- English language publication.
- Published between 2000 and 2024.
- Accessible full text.
- Qualitative or quantitative study design.

Exclusion:

- Letters to the editor and conference papers (due to perceived lower peer review standards and data quality).
- Retracted articles.

### 2.5 Quality Appraisal

The AACODS checklist was employed to assess the quality of the included articles in the study. This checklist comprises six questions categorized into “Authority,” “Accuracy,” “Objectivity,” “Date,” “Coverage,” and “Significance” [16]. Each question allowed three possible responses: yes, no, or can't answer, with points awarded for affirmative responses. Only studies scoring 9 and above were deemed eligible for inclusion in the study. This threshold was applied based on the final decision of the authors, as it was deemed suitable for inclusion/exclusion of the studies based on the content of the checklist items. The quality evaluation of the articles was independently conducted by two individuals.

### 2.6 Screening, Collating, Summarizing, and Thematic Analysis of the Data

During the process of screening the references, studies that were duplicates were removed, and the remaining ones underwent an evaluation based on their title and abstract. Those that did not correspond with the research objective were dismissed, and a comprehensive examination was conducted on the full text of the remaining studies. Only the studies that met the eligibility criteria were included in the final analysis. This entire process was carried out simultaneously by two researchers. In cases where there was a disagreement about the results of the process, other authors were consulted to finalize the screening process. Data that aligned with the study objective were extracted simultaneously by the two authors. A data extraction form was created using Microsoft Office Excel 2016 for the purpose of data collection. This form included sections such as authors, year of publication, country of origin, journal of publication, type of study, and context of the study.

After extracting data from eligible studies, we utilized MAXQDA version 10 for an inductive qualitative thematic analysis. Following Boyatzis's approach, the analysis unfolded in several steps [17]. Initially, all extracted data underwent multiple reviews and comparisons with the original texts to enhance familiarity. Subsequently, preliminary codes were identified based on the research question and desired outcomes. In the third step, an interpretive analysis of the initial codes was conducted, organizing them into subthemes and main themes. The subsequent phase involved a thorough review of these themes, allowing for their combination, refinement, separation, or elimination as needed. The final step encompassed defining and labeling themes along with their related sub‐themes, emphasizing content relevance. The thematic analysis was conducted collaboratively by two authors, and any disagreements arising during the process were resolved by consulting a third author, who provided the final decision.

## 3 Results

### 3.1 Systematic Review

**Figure 1:**
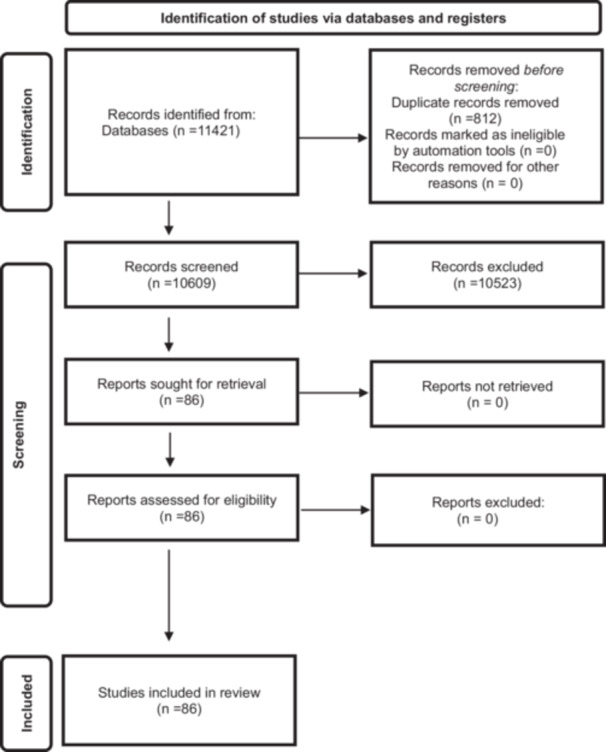
PRISMA diagram of the systematic review.

The search within the databases yielded 11,421 results, of which 812 were duplicates. The number of final studies was 86 after screening the titles, abstracts, and the text of the manuscripts (Figure 1). The studies were conducted from 2002 to 2024, and were published in various journals, carried out in multiple countries, including the United States, United Kingdom, Canada, Spain, Netherlands, Germany, Switzerland, Denmark, Nigeria, Jordan, Saudi Arabia, Norway, Republic of Korea, Austria, and some on a worldwide scale. The methodology of these studies was diverse, encompassing scoping reviews, cross‐sectional studies, qualitative studies, mixed methods studies, quantitative studies, systematic reviews, simulations, randomized controlled clinical trials, and case reports (Supporting Information S1: Appendix 1).

### 3.2 Quality Assessment of Final Articles

The evaluation of the final studies' quality revealed that the majority of studies achieved scores of 10, 11, or 12. This suggests a generally high standard of quality across the evaluated studies. The scores ranged from a minimum of 9 to a maximum of 12. Additionally, the level of bias present within the studies was deemed to be within acceptable limits. No study was excluded from this review due to a lack of quality (Supporting Information S2: Appendix 2).

### 3.3 Thematic Analysis

The thematic analysis of the data extracted from the final studies yielded 11 themes and 47 sub‐themes. The extracted themes included system design and integration, user characteristics, personalization, trust, quality, utility, satisfaction and acceptability, education and awareness, involvement, settings, and ethical‐legal issues (Table 2). Furthermore, each theme of factors exhibited a distinct and unique distribution of citations across the included studies. A more detailed description of the extracted themes is presented as follows.

- The system design and integration theme addresses the design and integration of DSS within healthcare systems. The results from the literature review highlighted that factors such as the fit of DSS with workflows, DSS technical dependencies and design, time, and transparency of processes are most frequently cited and play a significant role in the successful adoption of these systems.
- The theme of user characteristics refers to how different user profiles, including both physicians and patients, influence the acceptance and usage of DSS. According to the thematic analysis, various factors such as clinician gender, patient characteristics, and the overall demographic makeup impact how DSS is utilized.
- The personalization theme, which includes the sub‐themes of patient preferences and personalized approaches, emphasizes the importance of customizing DSS functionalities to align with both individual patient preferences and specific clinical needs.
- The trust theme presents that user trust in input data and the evidence base of DSS is a crucial factor for the adoption and effective use of these systems. Trust of users, validation, and provider mistrust and resistance are the subthemes identified within this context.
- The quality theme indicates that the quality of the DSS, the accuracy of its recommendations, and the quality of the services it supports significantly impact its utilization. Quality as a way to safety and effectiveness of the system are also factors influencing service utilization.
- The utility theme assesses the extent to which the DSS is useful for users and its impact on their performance. Relevance of clinical data, ease of use, and feasibility are the subthemes identified under this theme.
- The satisfaction and acceptability theme presents that user satisfaction and overall acceptance of DSS in healthcare systems directly affect its adoption.
- The education and awareness theme indicates that awareness of DSS functions, proper training, understanding its benefits, and access to precise clinical guidelines are essential for the adoption and effective use of DSS.
- The involvement theme presents that continuous stakeholder involvement is necessary to refine system functions and enhance its utilization. Ongoing collaboration can lead to more effective and widespread DSS solutions.
- The settings theme delineates that the specific geographical, social, and political conditions in which the DSS is implemented can significantly influence its level of utilization.
- The ethical‐legal issues theme indicates that privacy concerns, ethical dilemmas, patient satisfaction, and regulatory guidelines are also considerable factors affecting the effective utilization of DSS.

**Table 2:** Findings of the thematic analysis.

Theme	Sub‐theme	Reference	Percentage of citation
System design and integration	Fit of DSS with workflows	[18, 19, 20, 21, 22, 23, 24, 25, 26, 27, 28, 29, 30, 31, 32, 33, 34, 35, 36, 37, 38, 39, 40, 41, 42, 43, 44, 45, 46, 47]	53%
	DSS technical dependencies and design	[14, 18, 35, 40, 48, 49, 50]	
	Time	[25, 45, 46, 51, 52, 53, 54, 55, 56]	
	Format and wording	[57]	
	Technical complexity	[49, 58, 59]	
	Remote evaluation	[33, 60, 61]	
	Automatic labeling and analysis	[46, 60]	
	Therapy adjustment recommendations	[60]	
	Prioritization of patients	[60]	
	Visual communication	[23, 25, 46]	
	Evidence‐based	[23, 47]	
	Regular updates	[23, 29]	
	Transparency of processes	[43, 46, 49, 62]	
User characteristics	Clinician gender	[51]	6%
	Patient characteristics	[35, 51, 63, 64, 65, 66]	
Personalization	Patient preferences	[34, 54, 67, 68]	10%
	Personalized approach	[27, 34, 48, 50, 59, 67, 68, 69]	
Trust	Trust of users	[18, 22, 28, 29, 34, 39, 44, 53, 54, 60, 67, 70, 71, 72, 73]	22%
	Validation	[23, 54, 67]	
	Provider mistrust and resistance	[25, 26, 58]	
Quality	System quality	[24, 35, 47, 59, 63, 71, 73, 74, 75, 76, 77, 78, 79]	31%
	Information quality	[23, 75, 76, 80]	
	Service quality	[71, 75]	
	Effectiveness of the system	[9, 27, 33, 59, 81]	
	Perceived accuracy	[20, 46, 48, 52, 53, 79, 81, 82]	
	Reduction in unnecessary displacement	[60]	
	Effectiveness in identifying patients	[83]	
	Quality as a way to safety	[27, 63, 71, 81, 84]	
Utility	Relevance of clinical data	[22, 39, 57, 62, 67, 84]	44%
	Feasibility	[18, 20, 23, 25, 26, 28, 30, 31, 34, 35, 37, 47, 52, 55, 56, 59, 61, 62, 67, 70, 72, 73, 81, 85, 86, 87, 88, 89]	
	Ease of use	[25, 26, 32, 79, 90, 91]	
	Availability	[47, 87, 91, 92]	
Satisfaction and acceptability	User satisfaction	[24, 30, 48, 55, 57, 69, 73, 75, 78, 83, 85, 88, 91]	32%
	Acceptability	[14, 19, 26, 33, 38, 39, 49, 52, 53, 63, 67, 70, 81, 83, 89, 93]	
Education and awareness	Awareness	[20, 25, 29, 34, 42, 45, 53, 62, 66, 69, 86, 91, 92, 94, 95, 96]	37%
	Training	[14, 19, 25, 26, 40, 64, 66, 68, 77, 92, 97]	
	Perceived benefits	[32, 39, 40, 41, 45, 50, 53, 67, 69, 83, 85, 93, 98, 99, 100]	
	Understanding of the system	[14, 20, 57, 62, 70, 86, 91, 101, 102]	
	Access to right information	[74]	
Involvement	Continuous involvement of stakeholders	[20, 31, 36, 44, 45, 53, 56, 80, 82, 98]	19%
	Collaboration	[14, 20, 31, 33, 35, 36, 53, 77, 80, 94, 98, 99, 102]	
Settings	Implementation settings	[31, 35, 37, 53, 64, 76, 85, 94]	10%
	Unique barriers in low‐middle‐income countries	[47, 76, 85, 88]	
Ethical‐legal issues	Privacy	[56, 80]	13%
	Ethical challenges	[34, 46, 53, 68, 95]	
	Patient voluntary consent	[56]	
	Rules and regulations	[35, 36, 80, 87, 89]	

## 4 Discussion

The results of this study presented that 11 themes of factors are effective on the use of decision support systems within healthcare services: system design and integration, user characteristics, personalization, trust, quality, utility, satisfaction and acceptability, education and awareness, involvement, settings, and ethical‐legal issues. In this regard, as the results of the study presented, system design and integration emerged as the most frequently cited theme of factors influencing DSS adoption, accounting for 53% of the citations. Conversely, user characteristics constituted the theme with the least number of citations among the included studies, at 6%. This section of the study provides a comprehensive analysis of the results by examining the data in the context of previously published findings within the existing body of literature.

The findings of this study were aligned with previous reviews in the context, which had presented that the majority of studies in the literature focus on technological factors, while organizational factors receive less attention [11]. This phenomenon may be attributed to the fact that most studies in the existing literature originate from developed countries, rather than from developing countries, where the primary challenges are organizational rather than technological [103, 104, 105]. In this regard, a previously published quantitative systematic review revealed that the availability and quality of patient data are the most frequently cited technological factors influencing the adoption of DSS [12]. Meanwhile, in contrast to these findings, another systematic review presented that the majority of factors associated with the implementation of DSS pertain to organizational aspects. However, it also noted that technological factors have a significant impact, which aligns with the results of our study and the broader literature [14]. The discrepancies observed between this study and the existing literature are attributed to the narrower scope and fewer study inclusions in this study compared to our study and other reviews in the literature. Notably, the organizational factors highlighted in this study are predominantly reflected in our results, suggesting that the differences may stem from the scope and inclusivity of the studies [14].

Regarding the nature of factors influencing the adoption of DSS, another systematic review in the literature has reported that human and organizational factors exerted a negative influence on DSS utilization. Conversely, technological factors were found to have a neutral impact on DSS use [13]. However, given that the number of included studies and factors in this analysis is smaller compared to our current study, it cannot provide a comprehensive and precise representation of the nature of these factors and their influence on the adoption of DSS.

Policymakers can leverage the insights from this study to inform the development of policies and regulations supporting the adoption of DSS in healthcare. For instance, understanding the themes of system design and integration, quality, and ethical‐legal issues can help policymakers create frameworks that ensure DSS systems are integrated effectively into existing healthcare infrastructures, maintain high standards of quality, and address ethical and legal concerns [104, 106]. In developed countries, policies might focus on enhancing the technological infrastructure to support advanced DSS, while in developing countries, the emphasis could be on creating accessible and affordable solutions that meet local needs [103, 104, 105].

In order to enhance DSS implementation within healthcare ecosystems, healthcare practitioners can apply the study findings by considering how DSS can enhance their clinical decision‐making processes. For example, the themes of personalization, trust, satisfaction, and acceptability highlighted the importance of tailoring DSS to meet the specific needs of healthcare providers and patients, ensuring that these systems are trustworthy and user‐friendly. By integrating DSS into their workflows, practitioners can improve diagnostic accuracy, optimize treatment plans, and enhance patient safety [103, 106, 107].

This study's data can provide valuable implications in order to improve the structure and processes of CDSS within healthcare organizations. In this regard, CDSS can be applied in various ways, such as diagnostic assistance, medication optimization, and implementing clinical guidelines. By ensuring that CDSS aligns with the latest clinical guidelines and integrates patient‐specific data, healthcare organizations can reduce errors, improve patient outcomes, and enhance the efficiency of care delivery. The themes of education and awareness and involvement suggest that successful implementation of CDSS requires ongoing training and engagement of healthcare staff to ensure they are comfortable using these systems effectively [103, 104, 106].

### 4.1 Limitations and Implications

The research provided a comprehensive review of the multifarious factors influencing the utilization of DSS in healthcare. Adherence to the PRISMA guidelines ensured a methodical approach, enhancing the reliability of the findings. Furthermore, the thematic analysis offered nuanced insights into the complex interplay of technical, human, and policy‐related factors affecting DSS use. However, the research might be limited by the availability and selection of studies within the chosen databases. Despite systematic methods, thematic analysis could introduce interpretive bias from the researchers. In this regard, researchers' own personal attitudes and mindset could affect the process of conducting the analysis. Moreover, the fast‐paced advancements in DSS technology might outpace the findings presented in this review. Another limitation of this study was the absence of inter‐rater reliability calculations during the quality assessment process. The findings of the study are beneficial for healthcare policy makers and administrators who aim to implement DSS within their corresponding healthcare organizations. They can use these data to develop and implement strategies tailored to the factors identified in this study, with the aim of enhancing the utilization of DSS within their healthcare organizations. The findings can also be utilized by the researchers within the context, providing the necessary content on the context to conduct future research.

## 5 Conclusion

The study generated comprehensive data addressing the research questions by providing an extensive list of factors influencing the utilization of DSS in healthcare. Among these factors, system design and integration emerged as the most frequently cited theme affecting DSS adoption. The findings indicated that technological factors were referenced more often in the literature, whereas organizational factors received comparatively less attention. The study also demonstrated that the significance of these factors differs between developed and developing countries: developed countries tend to prioritize infrastructure, while developing countries focus on accessibility and affordability. Additionally, the successful implementation of CDSS requires continuous training and active engagement of healthcare personnel.

## Author Contributions

**Mohsen Khosravi:** conceptualization, investigation, and writing – original draft. **Zahra Zare:** writing – original draft, validation. **Seyyed Morteza Mojtabaeian:** investigation and resources. **Reyhane Izadi:** writing – original draft, supervision, and validation.

## Funding

The authors have nothing to report.

## Ethics Statement

The authors have nothing to report.

## Conflicts of Interest

The authors declare no conflicts of interest.

## Transparency Statement

R.I. and M.K. affirm that this manuscript is an honest, accurate, and transparent account of the study being reported; that no important aspects of the study have been omitted; and that any discrepancies from the study as planned have been explained.

## Supporting information

Supporting File 1

Supporting File 2

## Data Availability

The data that support the findings of this study are available from the corresponding author upon reasonable request.
