# Supplementary material for: A Systematic Review of Factors Affecting Utilization of Decision Support Systems: The Interplay Between Technology, Users, and the Healthcare Environment
Source: Health Sci Rep. 2026 Jun 12;9(6):e72665. doi: 10.1002/hsr2.72665 (PMC13263243; doi:10.1002/hsr2.72665)
Supplement: Supplementary file 1 — Supporting File 1 [file HSR2-9-e72665-s001.docx]

**Appendix 1. Bibliography of final studies.**

| No. | Author(s) | Year | Country | Journal | Study type | Context | Reference |
| --- | --- | --- | --- | --- | --- | --- | --- |
|  | Abell | 2023 | world | Implementation Science | scoping review | This review aimed to identify, categorize, and describe barriers and facilitators to CDSS implementation in hospital settings and map them to the NASSS framework. | [1] |
|  | Albahar | 2023 | Jordan | InHealthcare | cross-sectional study | This cross-sectional study examined healthcare professionals’ perceptions, barriers, and facilitators to adopting a CDSS for antibiotic prescribing in Jordanian hospitals. This study was conducted among healthcare professionals in Jordan’s two tertiary and teaching hospitals over four weeks (June–July 2021). | [2] |
|  | Alhodaib | 2020 | United Kingdom | JMIR diabetes | qualitative study | The purpose of this study was to conduct a feasibility project developing and evaluating a mobile clinical decision support system (CDSS) tool exclusively for health care providers to manage chronic kidney disease (CKD) in patients with DM. | [3] |
|  | Aljarboa | 2019 | Saudi Arabia | Asia-Pacific Conference on Computer Science and Data Engineering | qualitative study | This study extends the knowledge of CDSS use and assesses its acceptance by medical practitioners in Saudi Arabia. The research seeks to identify the factors through a qualitative interview technique that influence the acceptance of CDSS in healthcare in order to increase the knowledge as well as to provide more useful information that will contribute to CDSS adoption success. | [4] |
|  | Ankolekar | 2022 | Netherlands | Health Expectations | Mixed methods study | The aim of this study is to understand to what extent SDM is done in current practice and what clinicians need to improve it. | [5] |
|  | Bauer NS | 2016 | United States | Journal of the American Medical Informatics Association | quantitative study | The study sought to examine factors associated with clinician response to CDSS prompts as part of a larger, ongoing quality improvement effort to optimize CDSS use. | [6] |
|  | Bauer NS | 2014 | United States | Journal of the American Medical Informatics Association | quantitative study | The study wished to examine the attitudes and opinions of pediatric users' toward the Child Health Improvement through Computer Automation (CHICA) system, a computer decision support system linked to an electronic health record in four community pediatric clinics. | [7] |
|  | Belard | 2017 | world | Journal of clinical monitoring and computing | systematic review | The study conducted a systematic review of pertinent articles in the MEDLINE, US Department of Health and Human Services, Agency for Health Research and Quality, and US Food and Drug Administration databases, using a Boolean approach to combine terms germane to the discussion (clinical decision support, tools, systems, critical care, trauma, outcome, cost savings, NSQIP, APACHE, SOFA, ICU, and diagnostics). | [8] |
|  | Benrimoh | 2021 | Canada | BJPsych open | Simulation | Aifred is an artificial intelligence-powered clinical decision support system (CDSS) for the treatment of major depression. Here, the study explored the use of a simulation centre environment in evaluating the usability of Aifred, particularly its impact on the physician–patient interaction. | [9] |
|  | Bernasconi | 2019 | Nigeria | PloS one | cross-sectional study | The target area was the Federal State of Adamawa (Nigeria), a region recovering after the Boko Haram insurgency. The aim of this implementation research was to assess the improvement in terms of quality care offered after one year of utilization of the tool. | [10] |
|  | Besculides | 2023 | United States | JMIR Formative Research | qualitative study | This study aims to evaluate the implementation of an ML tool, Malnutrition Universal Screening Tool (MUST)–Plus, that predicts hospital patients at high risk for malnutrition and identify best implementation practices applicable to this and other ML-based CDSS. | [11] |
|  | Borum | 2018 | world | Nursing | literature review | A comprehensive literature review focused on the question: What are the barriers for nurse practitioners utilizing clinical decision support in the hospital setting? Nine studies conducted from 2011 to 2017 were the basis for this review, which identified 13 barriers for nurse practitioners utilizing clinical decision support in the hospital | [12] |
|  | Breitbart | 2020 | Denmark | PloS one | quantitative study | The primary objective of this feasibility study was to investigate patient experience and satisfaction in a primary care setting where general practitioners (GPs) used a visual clinical decision support system (CDSS) compared with standard consultations. Secondary objectives were diagnostic accuracy and length of consultation. | [13] |
|  | Catho | 2021 | Switzerland | Frontiers in Digital Health | Planning | In the context of the publicly funded COMPuterized Antibiotic Stewardship Study (COMPASS), the authors developed and implemented two CDSSs for antimicrobial prescriptions integrated into the in-house electronic health records of two public hospitals in Switzerland. | [14] |
|  | Caballero | 2017 | Spain | International journal of medical informatics | randomized controlled clinical trial | This paper presents Sinedie, a clinical decision support system designed to manage the treatment of patients with gestational diabetes. Sinedie aims to improve access to specialized healthcare assistance, to prevent patients from unnecessary displacements, to reduce the evaluation time per patient and to avoid gestational diabetes adverse outcomes. | [15] |
|  | Chadwick | 2017 | United Kingdom | HIV medicine | quantitative study | The objective of this study was to evaluate a risk-based prototype application to prompt HIV testing when patients undergo routine blood tests. | [16] |
|  | Chen | 2022 | world | Implementation Science Communications | qualitative systematic review | This qualitative systematic review seeks to synthesise healthcare provider experiences of CDS—exploring the barriers and enablers to implementing, using, evaluating, and sustaining chronic disease CDS systems. | [17] |
|  | Cho | 2022 | United States | JMIR human factors | quantitative study | This study presents an example application of a systematic evaluation method that uses clinician experts with human-computer interaction (HCI) expertise to evaluate the usability of an electronic clinical decision support (CDS) intervention prior to its deployment in a randomized controlled trial. | [18] |
|  | Choudhury | 2022 | United States | Frontiers in Digital Health | quantitative study | This study leverages validated questions to formulate an online survey and consequently explore cognitive human factors influencing clinicians' intention to use an AI-based Blood Utilization Calculator (BUC), an AI system embedded in the electronic health record that delivers data-driven personalized recommendations for the number of packed red blood cells to transfuse for a given patient. | [19] |
|  | Cresswell | 2017 | United Kingdom | Health Services Research | Qualitative case study | We undertook qualitative semi-structured interviews, non-participant observations of meetings and system use, and collected organizational documents over three time periods from six hospitals. Thematic analysis was initially undertaken within individual cases, followed by cross-case comparisons. | [20] |
|  | Daines | 2023 | United Kingdom | Journal of Asthma | qualitative study | We aimed to understand health professional views on the value of an asthma diagnosis CDSS and the barriers and facilitators for use in UK primary care. | [21] |
|  | El Mikati | 2020 | United States | Applied Clinical Informatics | quantitative study | Clinical decision support systems (CDSSs) provide clinicians with personalized reminders according to best evidence. One example is the Child Health Improvement through Computer Automation (CHICA) system, which, as we have previously shown, significantly improves screening for T2D. Given that the long-term success of any CDSS depends on its acceptability and its users' perceptions, we examined what clinicians think of the CHICA diabetes module | [22] |
|  | Emani | 2022 | world | JMIR cancer | literature review | In this paper, we review physicians’ perceptions of and satisfaction with an AI tool, Watson for Oncology, which is used for the treatment of cancer. | [23] |
|  | Fathauer | 2012 | United States | Applied clinical informatics | case report | The purpose of this case report is to describe the design, implementation, and initial evaluation of an HCV-specific CDSS while piloting data collection metrics and methods to be used in a larger study across multiple practices. | [24] |
|  | Fossum | 2012 | Norway | International Congress on Nursing Informatics | qualitative study | The aim was to describe the facilitators and barriers influencing the ability of nursing personnel to effectively use a CDSS for planning and treating pressure ulcers and malnutrition in nursing homes. | [25] |
|  | Fossum | 2011 | Norway | Applied clinical informatics | qualitative study | This study describes facilitators and barriers that impact the ability of nursing personnel to effectively use a clinical CDSS for planning and treating pressure ulcers (PUs) and malnutrition and for following the suggested risk assessment guidelines for the care of nursing home residents. | [26] |
|  | Fox | 2002 | United Kingdom | InProceedings of the AMIA Symposium | quantitative study | In future CDSS developers will be required (by the courts etc.) to acknowledge a "duty of care" covering all aspects of design, development and deployment.No single method will be sufficient for safe development and deployment; a range of techniques will be needed and used selectively. This paper is a contribution to discussion of quality, safety and legal liability issues in the medical informatics community. | [27] |
|  | Funer | 2023 | Germany | Journal of Medical Ethics | qualitative study | This study explores, from an ethical point of view, future healthcare professionals’ attitudes to potential changes of responsibility and decision-making authority when using ML-CDSS. | [28] |
|  | Genes | 2016 | United States | Applied clinical informatics | case report | To conduct an iterative usability and redesign process of a novel geriatric abdominal pain care CDSS. We hypothesized this process would result in the creation of more usable and favorable pain care interventions. | [29] |
|  | Ghorayeb | 2023 | United Kingdom | BMJ open | Mixed method | To develop and validate a questionnaire to assess the usability of clinical decision support systems (CDSS) and to assist in the early identification of usability issues that may impact patient safety and quality of care. | [30] |
|  | Goud | 2008 | Netherlands | eHealth Beyond the Horizon | quantitative study | The objective of this study was to investigate subjective usability of a guideline-based CDSS for outpatient cardiac rehabilitation. | [31] |
|  | Groenhof | 2019 | Netherlands | Heart Journal | project plan | The study set out to develop a real-time computerised decision support system (CDSS) embedded in the electronic health record (EHR) with information on risk factors, estimated risk, and guideline-based advice on treatment strategy in order to improve adherence to cardiovascular risk management (CVRM) guidelines with the ultimate aim of improving patient healthcare. | [32] |
|  | Grout | 2018 | United States | International journal of medical informatics | quantitative study | Long-term acceptability among computerized clinical decision support system (CDSS) users in pediatrics is unknown. We examine user acceptance patterns over six years of our continuous computerized CDSS integration and updates. | [33] |
|  | Hoelscher | 2020 | United States | Nursing | qualitative study | The purpose of this project was to develop a modular approach to integrate rapid deployment of clinical decision support for infectious diseases into the clinical workflow and evaluate the usability of the design | [34] |
|  | Jacobsohn | 2022 | United States | InHealthcare | Designing model | Existing fall screening tools have been poorly adopted due to ED staff/provider burden and lack of workflow integration. To address this, we developed an automated clinical decision support (CDS) system for identifying and referring older adult ED patients at risk of future falls. | [35] |
|  | Jaja | 2010 | United States | Journal of the National Medical Association | quantitative | This study assessed functionality and usability of the P4 program and identified problems in user-computer interaction in a sample of African American men. | [36] |
|  | Jeffries | 2021 | United Kingdom | PloS one | qualitative study | The study aimed to understand the factors that influenced the successful implementation and sustained use in primary care of a CDS system. | [37] |
|  | Jung | 2020 | Republic of Korea | JMIR Medical Informatics | Mixed methods study | In order to improve MDSS functionality, the study aimed to understand what factors users consider essential for the successful implementation of an MDSS into their clinical setting. This study followed the implementation process for an MDSS within a comprehensive EHR system and analyzed the relevant barriers and facilitators. | [38] |
|  | Kelsey | 2020 | United States | Journal of Primary Care & Community Health | quantitative study | The purpose of this study was to investigate primary care provider perceptions of utilizing CDSS alerts in the EMR to promote increased screening rates for breast cancer, cervical cancer, and colorectal cancer. | [39] |
|  | Khairat | 2018 | United States | JMIR medical informatics | quantitative study | The purpose of this paper was to conduct a critical review and task analysis of CDSS research and to develop a new framework for CDSS design in order to achieve user acceptance. | [40] |
|  | Kharbanda | 2015 | United States | eGEMs | two-year cluster-randomized clinical trial | The study has developed and piloted TeenBP— a web-based, electronic health record (EHR) linked system designed to improve recognition of prehypertension and hypertension in adolescents during primary care visits. | [41] |
|  | Kilsdonk | 2013 | Netherlands | Artificial intelligence in medicine | quantitative study | To assess whether a user-centred prototype clinical decision support system (CDSS) providing patient-specific advice better supports healthcare practitioners in terms of (a) types of usability problems detected and (b) effective and efficient retrieval of childhood cancer survivor's follow-up screening procedures compared to an expert-driven paper-based guideline. | [42] |
|  | Klarenbeek | 2020 | Netherlands | Biology | quantitative study | To successfully implement these CCDSSs in MDTMs, this study aims to: (a) identify barriers and facilitators for implementation for the use case of lung cancer; and (b) provide actionable findings for an implementation strategy. | [43] |
|  | Knitza | 2021 | Germany | Arthritis Research & Therapy | randomized clinical trial | The objectives were to compare the diagnostic accuracy of medical students, with and without the use of a DDSS, and the diagnostic accuracy of the DDSS system itself, regarding the typical rheumatic diseases and to analyze the user experience. | [44] |
|  | Kotsis | 2023 | Germany | BMC Medical Informatics and Decision Making | quantitative study | Nephrologists’ attitude towards any CDSS and potential CDSS features of interest, like adverse event prediction algorithms, is important for a successful implementation. This survey investigates nephrologists’ experiences with and expectations towards a useful CDSS for daily medical routine in the outpatient setting. | [45] |
|  | Kouladjian | 2020 | Canada | BMC Geriatrics | randomized clinical trial | This study aims to evaluate the implementation of a Computerised Clinical Decision Support System (CCDSS) called G-MEDSS© (Goal-directed Medication Review Electronic Decision Support System) in HMRs to deprescribe anticholinergic and sedative medications, and to assess the effect of deprescribing on clinical outcomes. | [46] |
|  | Kux | 2017 | world | InGMDS | qualitative study | This study sought to determine and evaluate barriers and facilitators to CDSS implementation and distribution. | [47] |
|  | Liberati | 2017 | United Kingdom | Implementation Science | qualitative study | This study examines the barriers and facilitators to the uptake of an evidence-based CDSS as perceived by diverse health professionals in hospitals at different stages of CDSS adoption. | [48] |
|  | Libiseller | 2020 | Austria | Contemporary Clinical Trials Communications | protocol | This study protocol describes a proof-of-concept study to investigate user acceptance, safety and efficacy of the GlucoTab@MobileCare system in participants receiving home health care. | [49] |
|  | Lobach | 2007 | United States | Studies in health technology and informatics | quantitative study | In this paper, the study reports the first implementation of a clinical decision support system using this new standard.We also report process measures and usability data concerning the system. | [50] |
|  | López | 2016 | Spain | Journal of medical systems | quantitative study | This paper aims to develop OphthalDSS, a totally new mobile DSS for red eye diseases diagnosis.Other important goal of this paper is to show the user experience results after OphthalDSS being used by medical students of the University of Valladolid. | [51] |
|  | Lugtenberg | 2015 | Netherlands | BMC medical informatics and decision making | qualitative study | The aim of this study was to identify the perceived barriers to using large-scale implemented CDSSs covering multiple disease areas in primary care. | [52] |
|  | Määttä | 2023 | Finland | JMIR Medical Informatics | quantitative study | The purpose of this study was to assess the performance, safety, and usability of a CDSS in a university hospital emergency department setting in Kuopio, Finland. | [53] |
|  | Madaras-Kelly | 2006 | United States | Journal of Managed Care Pharmacy | qualitative study | To determine the feasibility of a protocol-driven community pharmacy intervention that was designed to decrease broad-spectrum antimicrobial (BSA) use in patients with upper respiratory tract infections. | [54] |
|  | Marcolino | 2021 | Brazil | Journal of medical Internet research | mixed methods | The aim of this study is to develop a clinical decision support system (CDSS) for diabetes and hypertension management in primary care, to implement it in a resource-constrained region, and to evaluate its usability and health care practitioner satisfaction. | [55] |
|  | Markham | 2022 | United States | Frontiers in Public Health | quantitative study | We applied Implementation Mapping, a systematic planning framework that utilizes theory, empirical evidence, and community input, to adapt a theory-based, online decision support system, iCHAMPSS (CHoosing And Maintaining Effective Programs for Sex Education in Schools), to support underlying dissemination and implementation processes unique to Native communities. | [56] |
|  | Martinez-Garcia | 2021 | Spain | JMIR Medical Informatics | quantitative study | The aim of this study is to develop and evaluate a new tool (KNOWBED system) as a clinical decision support system to support scientific knowledge, enabling health care professionals to quickly carry out decision-making processes based on EBM during their routine clinical practice. | [57] |
|  | Nilsson | 2018 | Sweden | Contemporary Nurse | qualitative study | To describe home healthcare nurses’ experiences of the implementation and use of a new DSS, with a focus on how it influences decision making in everyday work practice. | [58] |
|  | Para Sanchez | 2023 | Netherlands | BMJ Health & Care Informatics | quantitative study | To design and develop an online Clinical Decision Support Systems (CDSS) tool “SLE-T2T”, and test its usability for the implementation of a T2T strategy in the management of patients with SLE. | [59] |
|  | Paulsen | 2019 | Norway | JMIR formative research | qualitative study | To investigate the possible implementation of the MyFood system in clinical practice, the aims of the study were (1) to identify current practice, routines, barriers, and facilitators of nutritional care; (2) to identify potential barriers and facilitators for the use of MyFood; and (3) to identify the key aspects of an implementation plan. | [60] |
|  | Paulsen | 2021 | Norway | BMC health services research | process evaluation study | The present study aimed to perform a process evaluation alongside the MyFood randomized controlled trial to evaluate the implementation plan and to understand implementation aspects to be able to prepare a scale-up of MyFood. | [61] |
|  | Petitgand | 2020 | Canada | InDigital Personalized Health and Medicine | qualitative study | This research examines the implementation of an AI-based decision support system (DSS) in the emergency department of a large Academic Health Center (AHC) in Canada, focusing specifically on the question of end-user adoption. | [62] |
|  | Pinar Manzanet | 2023 | Spain | Healthcare Technology Letters | Clinical study | This paper evaluates the CDSS miniQ for identifying potentially inappropriate prescribing in poly-medicated older adults and assesses the usability and acceptability of the system in health care professionals, patients, and caregivers. | [63] |
|  | Randell | 2010 | United Kingdom | International journal of medical informatics | qualitative study | The aim of this paper is to explore what nurses and NHS (National Health Service) managers working in NHS organisations in England perceive as the organisational features facilitating the introduction and successful use of CDSS. | [64] |
|  | Sambasivan | 2012 | Malaysia | BMC medical informatics and decision making | quantitative study | The main objectives of this study are to determine whether (1) the physician’s perceived professional autonomy, (2) involvement in the decision to implement CDSS and (3) the belief that CDSS will improve job performance increase the intention to adopt CDSS. Four hypotheses were formulated and tested. | [65] |
|  | Silveira | 2019 | Brazil | JMIR mHealth and uHealth | Mixed Methods | The goal of the research was to evaluate the feasibility, usability, and utility of a CDSS, TeleHAS (tele–hipertensão arterial sistêmica, or arterial hypertension system), in the care of patients with hypertension in the context of a primary care setting in a middle-income country. | [66] |
|  | Silvestri | 2022 | United States | JMIR Human Factors | Interview Study | We aimed to identify perceptions of predictive information in sepsis CDS systems based on clinicians’ past experiences, explore clinicians’ perceptions of a hypothetical sepsis CDS system, and identify the characteristics of a CDS system that would be helpful in promoting timely recognition and management of suspected sepsis in a multidisciplinary, team-based clinical setting. | [67] |
|  | Souza-Pereira | 2021 | Brazil | Journal of Biomedical Informatics | modeling | This study aims to propose a process model for evaluation of the quality in use characteristics of a CDSS to identify deficiencies that reduce its use by healthcare professionals. | [68] |
|  | Sukums | 2015 | Tanzania-Ghana | International journal of medical informatics | quantitative study | This study aimed to describe health workers’ acceptance and use of the eCDSS for maternal care in rural primary health care (PHC) facilities of Ghana and Tanzania and to identify factors affecting successful adoption of such a system. | [69] |
|  | Tokgoz | 2023 | world | BMC Medical Informatics and Decision Making | systematic review | The aim of this systematic review is to identify factors influencing the implementation of decision support systems for antibiotic prescription in hospitals. | [70] |
|  | Torres silva | 2018 | United States | HEALTH | observational study | The purpose of this paper is to present an evaluation of satisfaction and usability of a web-based CDSS called HADA. HADA was developed to improve antenatal healthcare by assisting in obstetric risk assessment. | [71] |
|  | Trafton | 2010 | United States | Pain Medicine | quantitative study | To develop and evaluate a clinical decision support system (CDSS) named Assessment and Treatment in Healthcare: Evidenced-Based Automation (ATHENA)-Opioid Therapy, which encourages safe and effective use of opioid therapy for chronic, noncancer pain. | [72] |
|  | Trivedi | 2009 | United States | BMC medical informatics and decision making | quantitative study | To examine the feasibility and effectiveness of implementation of a computerized decision support system for depression (CDSS-D) in routine public mental health care in Texas, fifteen study clinicians (thirteen physicians and two advanced nurse practitioners) participated across five sites, accruing over 300 outpatient visits on 168 patients. | [73] |
|  | Van Biesen | 2022 | Belgium | BMC Medical Informatics and Decision Making | Mixed Methods | We used a mix of quantitative and qualitative methods starting from Qsort methodology to explore expectations and perceptions of practicing physicians on the use of CDS incorporated in EHR. | [74] |
|  | Wickstrom | 2020 | Sweden | JMIR Human Factors | qualitative study | This study aims to describe health care staff’s experiences of engagement and barriers to and influencers of engagement when introducing a DDSS for wound management. | [75] |
|  | Zakane | 2017 | Sweden | Online Journal of Public Health Informatics | quantitative study | During the two years of the study period, it was apparent from reports that the CDSS was not used regularly in clinical practice. This study aimed to explore the reasons why HCWs failed to use the CDSS. | [76] |
|  | Zha | 2022 | China | BMC Medical Informatics and Decision Making | quantitative study | To investigate the determinant factors of nurses' acceptance of venous thromboembolism clinical decision support system with the modified Unified Theory of Acceptance and Use of Technology. | [77] |
|  | Zhai | 2022 | China | International Journal of Medical Informatics | qualitative study | Guided by the FITT framework, this study aims to explore barriers and facilitators to the implementation of a CDSS from the perspective of nurses. | [78] |

1. Abell, B., et al., *Identifying barriers and facilitators to successful implementation of computerized clinical decision support systems in hospitals: a NASSS framework-informed scoping review.* Implementation Science, 2023. **18**(1): p. 32.

2. Albahar, F., et al. *Healthcare Professionals’ Perceptions, Barriers, and Facilitators towards Adopting Computerised Clinical Decision Support Systems in Antimicrobial Stewardship in Jordanian Hospitals*. in *Healthcare*. 2023. MDPI.

3. Alhodaib, H.I., et al., *Mobile clinical decision support system for the management of diabetic patients with kidney complications in UK primary care settings: Mixed methods feasibility study.* JMIR diabetes, 2020. **5**(4): p. e19650.

4. Aljarboa, S. and S.J. Miah. *Investigating acceptance factors of Clinical Decision Support Systems in a developing country context*. in *2019 IEEE Asia-Pacific Conference on Computer Science and Data Engineering (CSDE)*. 2019. IEEE.

5. Ankolekar, A., et al., *Clinician perspectives on clinical decision support systems in lung cancer: Implications for shared decision‐making.* Health Expectations, 2022. **25**(4): p. 1342-1351.

6. Bauer, N.S., et al., *Experience with decision support system and comfort with topic predict clinicians’ responses to alerts and reminders.* Journal of the American Medical Informatics Association, 2016. **23**(e1): p. e125-e130.

7. Bauer, N.S., A.E. Carroll, and S.M. Downs, *Understanding the acceptability of a computer decision support system in pediatric primary care.* Journal of the American Medical Informatics Association, 2014. **21**(1): p. 146-153.

8. Belard, A., et al., *Precision diagnosis: a view of the clinical decision support systems (CDSS) landscape through the lens of critical care.* Journal of clinical monitoring and computing, 2017. **31**: p. 261-271.

9. Benrimoh, D., et al., *Using a simulation centre to evaluate preliminary acceptability and impact of an artificial intelligence-powered clinical decision support system for depression treatment on the physician–patient interaction.* BJPsych open, 2021. **7**(1): p. e22.

10. Bernasconi, A., et al., *Results from one-year use of an electronic Clinical Decision Support System in a post-conflict context: An implementation research.* PloS one, 2019. **14**(12): p. e0225634.

11. Besculides, M., et al., *Implementing a Machine Learning Screening Tool for Malnutrition: Insights From Qualitative Research Applicable to Other Machine Learning–Based Clinical Decision Support Systems.* JMIR Formative Research, 2023. **7**(1): p. e42262.

12. Borum, C., *Barriers for hospital-based nurse practitioners utilizing clinical decision support systems: a systematic review.* CIN: Computers, Informatics, Nursing, 2018. **36**(4): p. 177-182.

13. Breitbart, E.W., et al., *Improved patient satisfaction and diagnostic accuracy in skin diseases with a Visual Clinical Decision Support System—A feasibility study with general practitioners.* PloS one, 2020. **15**(7): p. e0235410.

14. Catho, G., et al., *How to develop and implement a computerized decision support system integrated for antimicrobial stewardship? Experiences from two Swiss hospital systems.* Frontiers in Digital Health, 2021. **2**: p. 583390.

15. Caballero-Ruiz, E., et al., *A web-based clinical decision support system for gestational diabetes: Automatic diet prescription and detection of insulin needs.* International journal of medical informatics, 2017. **102**: p. 35-49.

16. Chadwick, D., et al., *A feasibility study for a clinical decision support system prompting HIV testing.* HIV medicine, 2017. **18**(6): p. 435-439.

17. Chen, W., et al., *Barriers and enablers to implementing and using clinical decision support systems for chronic diseases: a qualitative systematic review and meta-aggregation.* Implementation Science Communications, 2022. **3**(1): p. 1-20.

18. Cho, H., et al., *Assessing the Usability of a Clinical Decision Support System: Heuristic Evaluation.* JMIR human factors, 2022. **9**(2): p. e31758.

19. Choudhury, A., *Factors influencing clinicians' willingness to use an AI-based clinical decision support system.* Frontiers in Digital Health, 2022. **4**: p. 920662.

20. Cresswell, K.M., et al., *Sustained user engagement in health information technology: the long road from implementation to system optimization of computerized physician order entry and clinical decision support systems for prescribing in hospitals in England.* Health Services Research, 2017. **52**(5): p. 1928-1957.

21. Daines, L., et al., *Clinician views on how clinical decision support systems can help diagnose asthma in primary care: a qualitative study.* Journal of Asthma, 2023(just-accepted): p. 1-13.

22. El Mikati, H.K., et al., *Clinician perceptions of a computerized decision support system for pediatric type 2 diabetes screening.* Applied Clinical Informatics, 2020. **11**(02): p. 350-355.

23. Emani, S., et al., *Physicians’ Perceptions of and Satisfaction With Artificial Intelligence in Cancer Treatment: A Clinical Decision Support System Experience and Implications for Low-Middle–Income Countries.* JMIR cancer, 2022. **8**(2): p. e31461.

24. Fathauer, L. and J. Meek, *Initial implementation and evaluation of a Hepatitis C treatment clinical decision support system (CDSS).* Applied clinical informatics, 2012. **3**(03): p. 337-348.

25. Fossum, M., et al. *The experiences of using a computerized decision support system*. in *NI 2012: 11th International Congress on Nursing Informatics, June 23-27, 2012, Montreal, Canada.* 2012. American Medical Informatics Association.

26. Fossum, M., et al., *An evaluation of the usability of a computerized decision support system for nursing homes.* Applied clinical informatics, 2011. **2**(04): p. 420-436.

27. Fox, J. and R. Thomson. *Clinical decision support systems: a discussion of quality, safety and legal liability issues*. in *Proceedings of the AMIA Symposium*. 2002. American Medical Informatics Association.

28. Funer, F., et al., *Responsibility and decision-making authority in using clinical decision support systems: an empirical-ethical exploration of German prospective professionals’ preferences and concerns.* Journal of Medical Ethics, 2024. **50**(1): p. 6-11.

29. Genes, N., et al., *Usability evaluation of a clinical decision support system for geriatric ED pain treatment.* Applied clinical informatics, 2016. **7**(01): p. 128-142.

30. Ghorayeb, A., et al., *Design and validation of a new Healthcare Systems Usability Scale (HSUS) for clinical decision support systems: a mixed-methods approach.* BMJ open, 2023. **13**(1): p. e065323.

31. Goud, R., et al. *Subjective usability of the CARDSS guideline-based decision support system*. in *MIE*. 2008.

32. Groenhof, T., et al., *A computerised decision support system for cardiovascular risk management ‘live’in the electronic health record environment: development, validation and implementation—the Utrecht Cardiovascular Cohort Initiative.* Netherlands Heart Journal, 2019. **27**: p. 435-442.

33. Grout, R.W., et al., *A six-year repeated evaluation of computerized clinical decision support system user acceptability.* International journal of medical informatics, 2018. **112**: p. 74-81.

34. Hoelscher, D. and S. McBride, *Usability and the rapid deployable infectious disease decision support system.* CIN: Computers, Informatics, Nursing, 2020. **38**(10): p. 490-499.

35. Jacobsohn, G.C., et al. *Collaborative design and implementation of a clinical decision support system for automated fall-risk identification and referrals in emergency departments*. in *Healthcare*. 2022. Elsevier.

36. Jaja, C., et al., *Usability evaluation of the interactive Personal Patient Profile-Prostate decision support system with African American men.* Journal of the National Medical Association, 2010. **102**(4): p. 290-302.

37. Jeffries, M., et al., *The implementation, use and sustainability of a clinical decision support system for medication optimisation in primary care: A qualitative evaluation.* PloS one, 2021. **16**(5): p. e0250946.

38. Jung, S.Y., et al., *Barriers and facilitators to implementation of medication decision support systems in electronic medical records: mixed methods approach based on structural equation modeling and qualitative analysis.* JMIR Medical Informatics, 2020. **8**(7): p. e18758.

39. Kelsey, E.A., et al., *Understanding user acceptance of clinical decision support systems to promote increased cancer screening rates in a primary care practice.* Journal of Primary Care & Community Health, 2020. **11**: p. 2150132720958832.

40. Khairat, S., et al., *Reasons for physicians not adopting clinical decision support systems: critical analysis.* JMIR medical informatics, 2018. **6**(2): p. e8912.

41. Kharbanda, E.O., et al., *TeenBP: development and piloting of an EHR-linked clinical decision support system to improve recognition of hypertension in adolescents.* eGEMs, 2015. **3**(2).

42. Kilsdonk, E., et al., *From an expert-driven paper guideline to a user-centred decision support system: a usability comparison study.* Artificial intelligence in medicine, 2013. **59**(1): p. 5-13.

43. Klarenbeek, S.E., et al., *Barriers and facilitators for implementation of a computerized clinical decision support system in lung cancer multidisciplinary team meetings—a qualitative assessment.* Biology, 2020. **10**(1): p. 9.

44. Knitza, J., et al., *Accuracy and usability of a diagnostic decision support system in the diagnosis of three representative rheumatic diseases: a randomized controlled trial among medical students.* Arthritis Research & Therapy, 2021. **23**(1): p. 1-10.

45. Kotsis, F., et al., *Expectation of clinical decision support systems: a survey study among nephrologist end-users.* BMC Medical Informatics and Decision Making, 2023. **23**(1): p. 239.

46. O’Donnell, L.K., et al., *Implementation of the Goal-directed Medication review Electronic Decision Support System (G-MEDSS)© into home medicines review: a protocol for a cluster-randomised clinical trial in older adults.* BMC geriatrics, 2020. **20**.

47. Kux, B.R., et al. *Factors Influencing the Implementation and Distribution of Clinical Decision Support Systems (CDSS)*. in *GMDS*. 2017.

48. Liberati, E.G., et al., *What hinders the uptake of computerized decision support systems in hospitals? A qualitative study and framework for implementation.* Implementation Science, 2017. **12**(1): p. 1-13.

49. Libiseller, A., et al., *Study protocol for assessing the user acceptance, safety and efficacy of a tablet-based workflow and decision support system with incorporated basal insulin algorithm for glycaemic management in participants with type 2 diabetes receiving home health care: a single-centre, open-label, uncontrolled proof-of-concept study.* Contemporary Clinical Trials Communications, 2020. **19**: p. 100620.

50. Lobach, D.F., et al., *Development, deployment and usability of a point-of-care decision support system for chronic disease management using the recently-approved HL7 decision support service standard.* Studies in health technology and informatics, 2007. **129**(Pt 2): p. 861-865.

51. López, M.M., et al., *A mobile decision support system for red eye diseases diagnosis: experience with medical students.* Journal of medical systems, 2016. **40**: p. 1-10.

52. Lugtenberg, M., et al., *Implementation of multiple-domain covering computerized decision support systems in primary care: a focus group study on perceived barriers.* BMC medical informatics and decision making, 2015. **15**: p. 1-11.

53. Määttä, J., et al., *Diagnostic Performance, Triage Safety, and Usability of a Clinical Decision Support System Within a University Hospital Emergency Department: Algorithm Performance and Usability Study.* JMIR Medical Informatics, 2023. **11**(1): p. e46760.

54. Madaras-Kelly, K.J., et al., *Experience with a clinical decision support system in community pharmacies to recommend narrow-spectrum antimicrobials, nonantimicrobial prescriptions, and OTC products to decrease broad-spectrum antimicrobial use.* Journal of Managed Care Pharmacy, 2006. **12**(5): p. 390-397.

55. Marcolino, M.S., et al., *Development and implementation of a decision support system to improve control of hypertension and diabetes in a resource-constrained area in Brazil: mixed methods study.* Journal of medical Internet research, 2021. **23**(1): p. e18872.

56. Markham, C.M., et al., *The Healthy Native Youth Implementation Toolbox: Using Implementation Mapping to adapt an online decision support system to promote culturally-relevant sexual health education for American Indian and Alaska Native youth.* Frontiers in Public Health, 2022. **10**: p. 889924.

57. Martinez-Garcia, A., et al., *A clinical decision support system (KNOWBED) to integrate scientific knowledge at the bedside: development and evaluation study.* JMIR Medical Informatics, 2021. **9**(3): p. e13182.

58. Nilsson, L. and C. Fagerström, *Decision-makers and mediators in a home healthcare digitisation process: nurses’ experiences of implementation and use of a decision support system.* Contemporary Nurse, 2018. **54**(4-5): p. 511-521.

59. Sanchez, A.R.P., et al., *Web-based eHealth Clinical Decision Support System as a tool for the treat-to-target management of patients with systemic lupus erythematosus: development and initial usability evaluation.* BMJ Health & Care Informatics, 2023. **30**(1).

60. Paulsen, M.M., et al., *Barriers and facilitators for implementing a decision support system to prevent and treat disease-related malnutrition in a hospital setting: qualitative study.* JMIR formative research, 2019. **3**(2): p. e11890.

61. Paulsen, M.M., C. Varsi, and L.F. Andersen, *Process evaluation of the implementation of a decision support system to prevent and treat disease-related malnutrition in a hospital setting.* BMC health services research, 2021. **21**(1): p. 1-13.

62. Petitgand, C., et al., *Investigating the barriers to physician adoption of an artificial intelligence-based decision support system in emergency care: an interpretative qualitative study*, in *Digital Personalized Health and Medicine*. 2020, IOS Press. p. 1001-1005.

63. Pinar Manzanet, J.M., et al., *Feasibility study of a clinical decision support system for polymedicated patients in primary care.* Healthcare Technology Letters, 2023.

64. Randell, R. and D. Dowding, *Organisational influences on nurses’ use of clinical decision support systems.* International journal of medical informatics, 2010. **79**(6): p. 412-421.

65. Sambasivan, M., et al., *Intention to adopt clinical decision support systems in a developing country: effect of Physician’s perceived professional autonomy, involvement and belief: a cross-sectional study.* BMC medical informatics and decision making, 2012. **12**(1): p. 1-8.

66. Silveira, D.V., et al., *Development and evaluation of a mobile decision support system for hypertension management in the primary care setting in Brazil: mixed-methods field study on usability, feasibility, and utility.* JMIR mHealth and uHealth, 2019. **7**(3): p. e9869.

67. Silvestri, J.A., et al., *Desired Characteristics of a Clinical Decision Support System for Early Sepsis Recognition: Interview Study Among Hospital-Based Clinicians.* JMIR Human Factors, 2022. **9**(4): p. e36976.

68. Pereira, L.M.R.d.S., *A process model for quality in use evaluation on clinical decision support systems.* 2022.

69. Sukums, F., et al., *Promising adoption of an electronic clinical decision support system for antenatal and intrapartum care in rural primary healthcare facilities in sub-Saharan Africa: The QUALMAT experience.* International journal of medical informatics, 2015. **84**(9): p. 647-657.

70. Tokgöz, P., J. Hafner, and C. Dockweiler, *Factors influencing the implementation of decision support systems for antibiotic prescription in hospitals: a systematic review.* BMC Medical Informatics and Decision Making, 2023. **23**(1): p. 1-12.

71. Silva, E.A.T., et al., *Evaluation of satisfaction and usability of a clinical decision support system (CDSS) targeted for early obstetric risk assessment and patient follow-up.* HEALTH, 2018: p. 3.

72. Trafton, J., et al., *Evaluation of the acceptability and usability of a decision support system to encourage safe and effective use of opioid therapy for chronic, noncancer pain by primary care providers.* Pain Medicine, 2010. **11**(4): p. 575-585.

73. Trivedi, M.H., et al., *Barriers to implementation of a computerized decision support system for depression: an observational report on lessons learned in.* BMC medical informatics and decision making, 2009. **9**(1): p. 1-9.

74. Van Biesen, W., et al., *An exploration of expectations and perceptions of practicing physicians on the implementation of computerized clinical decision support systems using a Qsort approach.* BMC Medical Informatics and Decision Making, 2022. **22**(1): p. 1-10.

75. Wickström, H., et al., *Health care Staff’s experiences of engagement when introducing a digital decision support system for wound management: qualitative study.* JMIR Human Factors, 2020. **7**(4): p. e23188.

76. Zakane, S.A., et al., *Opportunities and obstacles using a clinical decision support system for maternal care in Burkina Faso.* Online Journal of Public Health Informatics, 2017. **9**(2).

77. Zha, H., et al., *Acceptance of clinical decision support system to prevent venous thromboembolism among nurses: an extension of the UTAUT model.* BMC Medical Informatics and Decision Making, 2022. **22**(1): p. 221.

78. Zhai, Y., et al., *Barriers and facilitators to implementing a nursing clinical decision support system in a tertiary hospital setting: A qualitative study using the FITT framework.* International Journal of Medical Informatics, 2022. **166**: p. 104841.
