# Supplementary material for: A Systematic Review of Factors Affecting Utilization of Decision Support Systems: The Interplay Between Technology, Users, and the Healthcare Environment
Source: Health Sci Rep. 2026 Jun 12;9(6):e72665. doi: 10.1002/hsr2.72665 (PMC13263243; doi:10.1002/hsr2.72665)
Supplement: Supplementary file 2 — Supporting File 2 [file HSR2-9-e72665-s002.docx]

| **Appendix 2.** The result of quality assessment of selected articles based on the AACODS^[[1]](#footnote-1)^ checklist | | | | | | | | | |
| --- | --- | --- | --- | --- | --- | --- | --- | --- | --- |
| **Number** | **Author(s)/ Citation** | **Q1** | **Q2** | **Q3** | **Q4** | **Q5** | **Q6** | **Score** |  |
|  | Abell/[1] | 1 | 2 | 2 | 2 | 2 | 2 | 11 |  |
|  | Albahar/[2] | 2 | 2 | 2 | 2 | 2 | 2 | 12 |  |
|  | Alhodaib/[3] | 2 | 2 | 2 | 2 | 2 | 2 | 12 |  |
|  | Aljarboa/[4] | 0 | 2 | 2 | 2 | 2 | 2 | 10 |  |
|  | Ankolekar/[5] | 2 | 2 | 2 | 2 | 2 | 2 | 12 |  |
|  | Bauer NS/[6] | 2 | 2 | 1 | 2 | 0 | 2 | 9 |  |
|  | Bauer NS/[7] | 2 | 2 | 2 | 2 | 0 | 2 | 10 |  |
|  | Belard/[8] | 2 | 2 | 2 | 2 | 0 | 2 | 10 |  |
|  | Benrimoh/[9] | 2 | 2 | 2 | 2 | 2 | 2 | 12 |  |
|  | Bernasconi/[10] | 2 | 2 | 2 | 2 | 2 | 2 | 12 |  |
|  | Besculides/[11] | 2 | 2 | 2 | 2 | 2 | 1 | 11 |  |
|  | Borum/[12] | 2 | 1 | 2 | 2 | 0 | 2 | 9 |  |
|  | Breitbart/[13] | 2 | 2 | 2 | 2 | 2 | 2 | 12 |  |
|  | Catho/[14] | 2 | 2 | 2 | 2 | 2 | 2 | 12 |  |
|  | Caballero/[15] | 1 | 2 | 2 | 2 | 1 | 2 | 10 |  |
|  | Chadwick/[16] | 1 | 2 | 2 | 2 | 1 | 2 | 10 |  |
|  | Chen/[17] | 1 | 2 | 2 | 2 | 2 | 2 | 11 |  |
|  | Cho/[18] | 2 | 2 | 2 | 2 | 2 | 2 | 12 |  |
|  | Choudhury/[19] | 2 | 2 | 2 | 2 | 2 | 2 | 12 |  |
|  | Cresswell/[20] | 2 | 2 | 2 | 2 | 0 | 2 | 10 |  |
|  | Daines/[21] | 2 | 2 | 2 | 2 | 2 | 2 | 12 |  |
|  | El Mikati/[22] | 2 | 2 | 2 | 2 | 2 | 2 | 12 |  |
|  | Emani/[23] | 2 | 2 | 2 | 2 | 2 | 2 | 12 |  |
|  | Fathauer/[24] | 2 | 2 | 2 | 2 | 0 | 2 | 10 |  |
|  | Fossum/[25] | 2 | 2 | 2 | 2 | 0 | 2 | 10 |  |
|  | Fossum/[26] | 2 | 2 | 2 | 2 | 0 | 2 | 10 |  |
|  | Fox/[27] | 2 | 2 | 1 | 2 | 0 | 2 | 9 |  |
|  | Funer/[28] | 2 | 2 | 2 | 2 | 2 | 2 | 12 |  |
|  | Genes/[29] | 2 | 2 | 2 | 2 | 0 | 2 | 10 |  |
|  | Ghorayeb/[30] | 2 | 2 | 2 | 2 | 2 | 2 | 12 |  |
|  | Goud/[31] | 2 | 2 | 2 | 2 | 0 | 2 | 10 |  |
|  | Groenhof/[32] | 2 | 2 | 2 | 2 | 2 | 2 | 12 |  |
|  | Grout/[33] | 2 | 2 | 2 | 2 | 2 | 2 | 12 |  |
|  | Hoelscher/[34] | 2 | 2 | 2 | 2 | 2 | 2 | 12 |  |
|  | Jacobsohn/[35] | 2 | 2 | 2 | 2 | 2 | 2 | 12 |  |
|  | Jaja/[36] | 1 | 2 | 2 | 2 | 0 | 2 | 9 |  |
|  | Jeffries/[37] | 2 | 2 | 2 | 2 | 2 | 2 | 12 |  |
|  | Jung/[38] | 2 | 2 | 2 | 2 | 2 | 2 | 12 |  |
|  | Kelsey/[39] | 2 | 2 | 2 | 2 | 2 | 2 | 12 |  |
|  | Khairat/[40] | 2 | 2 | 2 | 2 | 2 | 2 | 12 |  |
|  | Kharbanda/[41] | 2 | 2 | 2 | 2 | 0 | 2 | 10 |  |
|  | Kilsdonk/[42] | 2 | 2 | 2 | 2 | 0 | 2 | 10 |  |
|  | Klarenbeek/[43] | 2 | 2 | 2 | 2 | 2 | 2 | 12 |  |
|  | Knitza/[44] | 2 | 2 | 2 | 2 | 2 | 2 | 12 |  |
|  | Kotsis/[45] | 2 | 2 | 2 | 2 | 2 | 2 | 12 |  |
|  | Kouladjian/[46] | 2 | 2 | 2 | 2 | 2 | 2 | 12 |  |
|  | Kux/[47] | 2 | 2 | 1 | 2 | 0 | 2 | 9 |  |
|  | Liberati/[48] | 2 | 2 | 2 | 2 | 1 | 2 | 11 |  |
|  | Libiseller/[49] | 2 | 2 | 2 | 2 | 1 | 2 | 11 |  |
|  | Lobach/[50] | 2 | 2 | 2 | 2 | 0 | 2 | 10 |  |
|  | López/[51] | 2 | 2 | 1 | 2 | 0 | 2 | 9 |  |
|  | Lugtenberg/[52] | 2 | 2 | 2 | 2 | 0 | 2 | 10 |  |
|  | Määttä/[53] | 2 | 2 | 2 | 2 | 1 | 2 | 11 |  |
|  | Madaras-Kelly/[54] | 2 | 2 | 2 | 2 | 0 | 2 | 10 |  |
|  | Marcolino/[55] | 2 | 2 | 2 | 2 | 1 | 2 | 11 |  |
|  | Markham/[56] | 2 | 2 | 2 | 2 | 2 | 2 | 12 |  |
|  | Martinez-Garcia/[57] | 2 | 2 | 2 | 2 | 1 | 2 | 11 |  |
|  | Nilsson/[58] | 2 | 2 | 2 | 2 | 1 | 2 | 11 |  |
|  | Parra Sanchez/[59] | 2 | 2 | 2 | 2 | 2 | 2 | 12 |  |
|  | Paulsen/[60] | 2 | 2 | 2 | 2 | 1 | 2 | 11 |  |
|  | Paulsen/[61] | 2 | 2 | 2 | 2 | 2 | 2 | 12 |  |
|  | Petitgand/[62] | 2 | 2 | 2 | 2 | 2 | 2 | 12 |  |
|  | Pinar Manzanet/[63] | 2 | 2 | 2 | 2 | 2 | 2 | 12 |  |
|  | Randell/[64] | 2 | 2 | 2 | 2 | 0 | 2 | 10 |  |
|  | Sambasivan/[65] | 2 | 2 | 2 | 1 | 0 | 2 | 9 |  |
|  | Silveira/[66] | 2 | 2 | 2 | 2 | 1 | 2 | 11 |  |
|  | Silvestri/[67] | 2 | 2 | 2 | 2 | 2 | 2 | 12 |  |
|  | Souza-Pereira/[68] | 2 | 2 | 2 | 2 | 2 | 2 | 12 |  |
|  | Sukums/[69] | 2 | 2 | 2 | 2 | 0 | 2 | 10 |  |
|  | Tokgoz/[70] | 2 | 2 | 2 | 2 | 2 | 2 | 12 |  |
|  | Torres silva/[71] | 2 | 2 | 2 | 2 | 1 | 2 | 11 |  |
|  | Trafton/[72] | 2 | 2 | 2 | 2 | 0 | 2 | 10 |  |
|  | Trivedi/[73] | 2 | 2 | 2 | 2 | 1 | 2 | 11 |  |
|  | Van Biesen/[74] | 2 | 2 | 2 | 2 | 2 | 2 | 12 |  |
|  | Wickstrom/[75] | 2 | 2 | 1 | 2 | 1 | 2 | 10 |  |
|  | Zakane/[76] | 2 | 2 | 2 | 2 | 0 | 2 | 10 |  |
|  | Zha/[77] | 2 | 2 | 2 | 2 | 2 | 2 | 12 |  |
|  | Zhai/[78] | 2 | 2 | 2 | 2 | 1 | 2 | 11 |  |
| Scoring AACODS checklist questions: Yes =2, Can’t Tell= 1, No = 0 | | | | | | | | | |
| Q1: Authority: Is the author or source of the information reputable and trustworthy?  Q2: Accuracy: Is the information reliable, truthful, and correct?  Q3: Coverage: Does the information cover the topic comprehensively and sufficiently?  Q4: Objectivity: Is the information presented in an unbiased and balanced way?  Q5: Date: Is the information current and up-to-date?  Q6: Significance: Is the information relevant, important, and valuable to the topic? | | | | | | | | | |

**References:**

1. Abell, B., et al., *Identifying barriers and facilitators to successful implementation of computerized clinical decision support systems in hospitals: a NASSS framework-informed scoping review.* Implementation Science, 2023. **18**(1): p. 32.

2. Albahar, F., et al. *Healthcare Professionals’ Perceptions, Barriers, and Facilitators towards Adopting Computerised Clinical Decision Support Systems in Antimicrobial Stewardship in Jordanian Hospitals*. in *Healthcare*. 2023. MDPI.

3. Alhodaib, H.I., et al., *Mobile clinical decision support system for the management of diabetic patients with kidney complications in UK primary care settings: Mixed methods feasibility study.* JMIR diabetes, 2020. **5**(4): p. e19650.

4. Aljarboa, S. and S.J. Miah. *Investigating acceptance factors of Clinical Decision Support Systems in a developing country context*. in *2019 IEEE Asia-Pacific Conference on Computer Science and Data Engineering (CSDE)*. 2019. IEEE.

5. Ankolekar, A., et al., *Clinician perspectives on clinical decision support systems in lung cancer: Implications for shared decision‐making.* Health Expectations, 2022. **25**(4): p. 1342-1351.

6. Bauer, N.S., et al., *Experience with decision support system and comfort with topic predict clinicians’ responses to alerts and reminders.* Journal of the American Medical Informatics Association, 2016. **23**(e1): p. e125-e130.

7. Bauer, N.S., A.E. Carroll, and S.M. Downs, *Understanding the acceptability of a computer decision support system in pediatric primary care.* Journal of the American Medical Informatics Association, 2014. **21**(1): p. 146-153.

8. Belard, A., et al., *Precision diagnosis: a view of the clinical decision support systems (CDSS) landscape through the lens of critical care.* Journal of clinical monitoring and computing, 2017. **31**: p. 261-271.

9. Benrimoh, D., et al., *Using a simulation centre to evaluate preliminary acceptability and impact of an artificial intelligence-powered clinical decision support system for depression treatment on the physician–patient interaction.* BJPsych open, 2021. **7**(1): p. e22.

10. Bernasconi, A., et al., *Results from one-year use of an electronic Clinical Decision Support System in a post-conflict context: An implementation research.* PloS one, 2019. **14**(12): p. e0225634.

11. Besculides, M., et al., *Implementing a Machine Learning Screening Tool for Malnutrition: Insights From Qualitative Research Applicable to Other Machine Learning–Based Clinical Decision Support Systems.* JMIR Formative Research, 2023. **7**(1): p. e42262.

12. Borum, C., *Barriers for hospital-based nurse practitioners utilizing clinical decision support systems: a systematic review.* CIN: Computers, Informatics, Nursing, 2018. **36**(4): p. 177-182.

13. Breitbart, E.W., et al., *Improved patient satisfaction and diagnostic accuracy in skin diseases with a Visual Clinical Decision Support System—A feasibility study with general practitioners.* PloS one, 2020. **15**(7): p. e0235410.

14. Catho, G., et al., *How to develop and implement a computerized decision support system integrated for antimicrobial stewardship? Experiences from two Swiss hospital systems.* Frontiers in Digital Health, 2021. **2**: p. 583390.

15. Caballero-Ruiz, E., et al., *A web-based clinical decision support system for gestational diabetes: Automatic diet prescription and detection of insulin needs.* International journal of medical informatics, 2017. **102**: p. 35-49.

16. Chadwick, D., et al., *A feasibility study for a clinical decision support system prompting HIV testing.* HIV medicine, 2017. **18**(6): p. 435-439.

17. Chen, W., et al., *Barriers and enablers to implementing and using clinical decision support systems for chronic diseases: a qualitative systematic review and meta-aggregation.* Implementation Science Communications, 2022. **3**(1): p. 1-20.

18. Cho, H., et al., *Assessing the Usability of a Clinical Decision Support System: Heuristic Evaluation.* JMIR human factors, 2022. **9**(2): p. e31758.

19. Choudhury, A., *Factors influencing clinicians' willingness to use an AI-based clinical decision support system.* Frontiers in Digital Health, 2022. **4**: p. 920662.

20. Cresswell, K.M., et al., *Sustained user engagement in health information technology: the long road from implementation to system optimization of computerized physician order entry and clinical decision support systems for prescribing in hospitals in England.* Health Services Research, 2017. **52**(5): p. 1928-1957.

21. Daines, L., et al., *Clinician views on how clinical decision support systems can help diagnose asthma in primary care: a qualitative study.* Journal of Asthma, 2023(just-accepted): p. 1-13.

22. El Mikati, H.K., et al., *Clinician perceptions of a computerized decision support system for pediatric type 2 diabetes screening.* Applied Clinical Informatics, 2020. **11**(02): p. 350-355.

23. Emani, S., et al., *Physicians’ Perceptions of and Satisfaction With Artificial Intelligence in Cancer Treatment: A Clinical Decision Support System Experience and Implications for Low-Middle–Income Countries.* JMIR cancer, 2022. **8**(2): p. e31461.

24. Fathauer, L. and J. Meek, *Initial implementation and evaluation of a Hepatitis C treatment clinical decision support system (CDSS).* Applied clinical informatics, 2012. **3**(03): p. 337-348.

25. Fossum, M., et al. *The experiences of using a computerized decision support system*. in *NI 2012: 11th International Congress on Nursing Informatics, June 23-27, 2012, Montreal, Canada.* 2012. American Medical Informatics Association.

26. Fossum, M., et al., *An evaluation of the usability of a computerized decision support system for nursing homes.* Applied clinical informatics, 2011. **2**(04): p. 420-436.

27. Fox, J. and R. Thomson. *Clinical decision support systems: a discussion of quality, safety and legal liability issues*. in *Proceedings of the AMIA Symposium*. 2002. American Medical Informatics Association.

28. Funer, F., et al., *Responsibility and decision-making authority in using clinical decision support systems: an empirical-ethical exploration of German prospective professionals’ preferences and concerns.* Journal of Medical Ethics, 2024. **50**(1): p. 6-11.

29. Genes, N., et al., *Usability evaluation of a clinical decision support system for geriatric ED pain treatment.* Applied clinical informatics, 2016. **7**(01): p. 128-142.

30. Ghorayeb, A., et al., *Design and validation of a new Healthcare Systems Usability Scale (HSUS) for clinical decision support systems: a mixed-methods approach.* BMJ open, 2023. **13**(1): p. e065323.

31. Goud, R., et al. *Subjective usability of the CARDSS guideline-based decision support system*. in *MIE*. 2008.

32. Groenhof, T., et al., *A computerised decision support system for cardiovascular risk management ‘live’in the electronic health record environment: development, validation and implementation—the Utrecht Cardiovascular Cohort Initiative.* Netherlands Heart Journal, 2019. **27**: p. 435-442.

33. Grout, R.W., et al., *A six-year repeated evaluation of computerized clinical decision support system user acceptability.* International journal of medical informatics, 2018. **112**: p. 74-81.

34. Hoelscher, D. and S. McBride, *Usability and the rapid deployable infectious disease decision support system.* CIN: Computers, Informatics, Nursing, 2020. **38**(10): p. 490-499.

35. Jacobsohn, G.C., et al. *Collaborative design and implementation of a clinical decision support system for automated fall-risk identification and referrals in emergency departments*. in *Healthcare*. 2022. Elsevier.

36. Jaja, C., et al., *Usability evaluation of the interactive Personal Patient Profile-Prostate decision support system with African American men.* Journal of the National Medical Association, 2010. **102**(4): p. 290-302.

37. Jeffries, M., et al., *The implementation, use and sustainability of a clinical decision support system for medication optimisation in primary care: A qualitative evaluation.* PloS one, 2021. **16**(5): p. e0250946.

38. Jung, S.Y., et al., *Barriers and facilitators to implementation of medication decision support systems in electronic medical records: mixed methods approach based on structural equation modeling and qualitative analysis.* JMIR Medical Informatics, 2020. **8**(7): p. e18758.

39. Kelsey, E.A., et al., *Understanding user acceptance of clinical decision support systems to promote increased cancer screening rates in a primary care practice.* Journal of Primary Care & Community Health, 2020. **11**: p. 2150132720958832.

40. Khairat, S., et al., *Reasons for physicians not adopting clinical decision support systems: critical analysis.* JMIR medical informatics, 2018. **6**(2): p. e8912.

41. Kharbanda, E.O., et al., *TeenBP: development and piloting of an EHR-linked clinical decision support system to improve recognition of hypertension in adolescents.* eGEMs, 2015. **3**(2).

42. Kilsdonk, E., et al., *From an expert-driven paper guideline to a user-centred decision support system: a usability comparison study.* Artificial intelligence in medicine, 2013. **59**(1): p. 5-13.

43. Klarenbeek, S.E., et al., *Barriers and facilitators for implementation of a computerized clinical decision support system in lung cancer multidisciplinary team meetings—a qualitative assessment.* Biology, 2020. **10**(1): p. 9.

44. Knitza, J., et al., *Accuracy and usability of a diagnostic decision support system in the diagnosis of three representative rheumatic diseases: a randomized controlled trial among medical students.* Arthritis Research & Therapy, 2021. **23**(1): p. 1-10.

45. Kotsis, F., et al., *Expectation of clinical decision support systems: a survey study among nephrologist end-users.* BMC Medical Informatics and Decision Making, 2023. **23**(1): p. 239.

46. O’Donnell, L.K., et al., *Implementation of the Goal-directed Medication review Electronic Decision Support System (G-MEDSS)© into home medicines review: a protocol for a cluster-randomised clinical trial in older adults.* BMC geriatrics, 2020. **20**.

47. Kux, B.R., et al. *Factors Influencing the Implementation and Distribution of Clinical Decision Support Systems (CDSS)*. in *GMDS*. 2017.

48. Liberati, E.G., et al., *What hinders the uptake of computerized decision support systems in hospitals? A qualitative study and framework for implementation.* Implementation Science, 2017. **12**(1): p. 1-13.

49. Libiseller, A., et al., *Study protocol for assessing the user acceptance, safety and efficacy of a tablet-based workflow and decision support system with incorporated basal insulin algorithm for glycaemic management in participants with type 2 diabetes receiving home health care: a single-centre, open-label, uncontrolled proof-of-concept study.* Contemporary Clinical Trials Communications, 2020. **19**: p. 100620.

50. Lobach, D.F., et al., *Development, deployment and usability of a point-of-care decision support system for chronic disease management using the recently-approved HL7 decision support service standard.* Studies in health technology and informatics, 2007. **129**(Pt 2): p. 861-865.

51. López, M.M., et al., *A mobile decision support system for red eye diseases diagnosis: experience with medical students.* Journal of medical systems, 2016. **40**: p. 1-10.

52. Lugtenberg, M., et al., *Implementation of multiple-domain covering computerized decision support systems in primary care: a focus group study on perceived barriers.* BMC medical informatics and decision making, 2015. **15**: p. 1-11.

53. Määttä, J., et al., *Diagnostic Performance, Triage Safety, and Usability of a Clinical Decision Support System Within a University Hospital Emergency Department: Algorithm Performance and Usability Study.* JMIR Medical Informatics, 2023. **11**(1): p. e46760.

54. Madaras-Kelly, K.J., et al., *Experience with a clinical decision support system in community pharmacies to recommend narrow-spectrum antimicrobials, nonantimicrobial prescriptions, and OTC products to decrease broad-spectrum antimicrobial use.* Journal of Managed Care Pharmacy, 2006. **12**(5): p. 390-397.

55. Marcolino, M.S., et al., *Development and implementation of a decision support system to improve control of hypertension and diabetes in a resource-constrained area in Brazil: mixed methods study.* Journal of medical Internet research, 2021. **23**(1): p. e18872.

56. Markham, C.M., et al., *The Healthy Native Youth Implementation Toolbox: Using Implementation Mapping to adapt an online decision support system to promote culturally-relevant sexual health education for American Indian and Alaska Native youth.* Frontiers in Public Health, 2022. **10**: p. 889924.

57. Martinez-Garcia, A., et al., *A clinical decision support system (KNOWBED) to integrate scientific knowledge at the bedside: development and evaluation study.* JMIR Medical Informatics, 2021. **9**(3): p. e13182.

58. Nilsson, L. and C. Fagerström, *Decision-makers and mediators in a home healthcare digitisation process: nurses’ experiences of implementation and use of a decision support system.* Contemporary Nurse, 2018. **54**(4-5): p. 511-521.

59. Sanchez, A.R.P., et al., *Web-based eHealth Clinical Decision Support System as a tool for the treat-to-target management of patients with systemic lupus erythematosus: development and initial usability evaluation.* BMJ Health & Care Informatics, 2023. **30**(1).

60. Paulsen, M.M., et al., *Barriers and facilitators for implementing a decision support system to prevent and treat disease-related malnutrition in a hospital setting: qualitative study.* JMIR formative research, 2019. **3**(2): p. e11890.

61. Paulsen, M.M., C. Varsi, and L.F. Andersen, *Process evaluation of the implementation of a decision support system to prevent and treat disease-related malnutrition in a hospital setting.* BMC health services research, 2021. **21**(1): p. 1-13.

62. Petitgand, C., et al., *Investigating the barriers to physician adoption of an artificial intelligence-based decision support system in emergency care: an interpretative qualitative study*, in *Digital Personalized Health and Medicine*. 2020, IOS Press. p. 1001-1005.

63. Pinar Manzanet, J.M., et al., *Feasibility study of a clinical decision support system for polymedicated patients in primary care.* Healthcare Technology Letters, 2023.

64. Randell, R. and D. Dowding, *Organisational influences on nurses’ use of clinical decision support systems.* International journal of medical informatics, 2010. **79**(6): p. 412-421.

65. Sambasivan, M., et al., *Intention to adopt clinical decision support systems in a developing country: effect of Physician’s perceived professional autonomy, involvement and belief: a cross-sectional study.* BMC medical informatics and decision making, 2012. **12**(1): p. 1-8.

66. Silveira, D.V., et al., *Development and evaluation of a mobile decision support system for hypertension management in the primary care setting in Brazil: mixed-methods field study on usability, feasibility, and utility.* JMIR mHealth and uHealth, 2019. **7**(3): p. e9869.

67. Silvestri, J.A., et al., *Desired Characteristics of a Clinical Decision Support System for Early Sepsis Recognition: Interview Study Among Hospital-Based Clinicians.* JMIR Human Factors, 2022. **9**(4): p. e36976.

68. Pereira, L.M.R.d.S., *A process model for quality in use evaluation on clinical decision support systems.* 2022.

69. Sukums, F., et al., *Promising adoption of an electronic clinical decision support system for antenatal and intrapartum care in rural primary healthcare facilities in sub-Saharan Africa: The QUALMAT experience.* International journal of medical informatics, 2015. **84**(9): p. 647-657.

70. Tokgöz, P., J. Hafner, and C. Dockweiler, *Factors influencing the implementation of decision support systems for antibiotic prescription in hospitals: a systematic review.* BMC Medical Informatics and Decision Making, 2023. **23**(1): p. 1-12.

71. Silva, E.A.T., et al., *Evaluation of satisfaction and usability of a clinical decision support system (CDSS) targeted for early obstetric risk assessment and patient follow-up.* HEALTH, 2018: p. 3.

72. Trafton, J., et al., *Evaluation of the acceptability and usability of a decision support system to encourage safe and effective use of opioid therapy for chronic, noncancer pain by primary care providers.* Pain Medicine, 2010. **11**(4): p. 575-585.

73. Trivedi, M.H., et al., *Barriers to implementation of a computerized decision support system for depression: an observational report on lessons learned in.* BMC medical informatics and decision making, 2009. **9**(1): p. 1-9.

74. Van Biesen, W., et al., *An exploration of expectations and perceptions of practicing physicians on the implementation of computerized clinical decision support systems using a Qsort approach.* BMC Medical Informatics and Decision Making, 2022. **22**(1): p. 1-10.

75. Wickström, H., et al., *Health care Staff’s experiences of engagement when introducing a digital decision support system for wound management: qualitative study.* JMIR Human Factors, 2020. **7**(4): p. e23188.

76. Zakane, S.A., et al., *Opportunities and obstacles using a clinical decision support system for maternal care in Burkina Faso.* Online Journal of Public Health Informatics, 2017. **9**(2).

77. Zha, H., et al., *Acceptance of clinical decision support system to prevent venous thromboembolism among nurses: an extension of the UTAUT model.* BMC Medical Informatics and Decision Making, 2022. **22**(1): p. 221.

78. Zhai, Y., et al., *Barriers and facilitators to implementing a nursing clinical decision support system in a tertiary hospital setting: A qualitative study using the FITT framework.* International Journal of Medical Informatics, 2022. **166**: p. 104841.

1. Accuracy, Coverage, Objectivity, Date, Significance [↑](#footnote-ref-1)
